# The complete chloroplast genome of a purple Ethiopian rape (*Brassica carinata*: Brassicaceae) from Guizhou Province, China and its phylogenetic analysis

**DOI:** 10.1080/23802359.2021.1926365

**Published:** 2021-06-03

**Authors:** Bin Zhu, Zuomin Gao, Fang Qian, Xiaoli Yang, Xianju Lv, Mengxian Cai

**Affiliations:** School of Life Sciences, Guizhou Normal University, Guiyang, People’s Republic of China

**Keywords:** *Brassica carinata*, chloroplast genome, phylogenetic analysis

## Abstract

*Brassica carinata* A. Braun (Ethiopian rape), which was derived from the interspecific hybridization between *B. nigra* and *B. oleracea*, is used as both an oilseed and a leafy vegetable. The complete chloroplast (cp) genome of a purple *B. carinata* was obtained. This cp genome has a typical quadripartite structure and is 153,641 bp in length. The GC content of the cp genome is 36.39%. A total of 113 genes were predicted on this cp genome, including 79 protein coding, 4 rRNA, and 30 tRNA genes. Among these genes, 18 genes were duplicated (7 tRNAs, 4 rRNAs, and 7 protein coding genes). Sixty-eight SSR loci, including 11 compound SSRs, were identified in this cp genome by MISA. The phylogenetic tree analysis fully resolved *B. carinata* in a clade with *B. nigra*. This study provides important information for future evolution, genetic and molecular biology studies of *B. carinata*.

*Brassica carinata*, referred to by the common names Ethiopian rape, is an amphidiploid species derived from the diploid species *B. nigra* and *B. oleracea* (Nagaharu 1935). It is believed to have originated in Ethiopia (Gómez-Campo and Prakash 1999), where it is used as both an oilseed and a leafy vegetable. Seed oil from *B. carinata* has industrial applications due to its high erucic or linolenic acid contents (Warwick et al. 2006).

A deep purple *B. carinata* (accession BC-PL1) was gathered from Longwen mountain (26°23′11″N, 106°38′32″E), Guizhou province, China, and the voucher specimen was deposited in the herbarium of Guizhou Normal University (URL: https://sjxy.gznu.edu.cn/, Qun Feng, feng18085615616@163.com) under the voucher number: ZB20201011. The total DNA of *B. carinata* was extracted by CTAB method, and the DNA libraries were prepared and sequenced using the Illumina HiSeq 4000 and PacBio Sequel platform (Illumina Inc. San Diego, CA). The cp genome was assembled using a *de novo* strategy according to the work of Du et al. (2020). The annotated cp genome of *B. carinata* is publicly available in GenBank (accession number of MW628493).

The cp genome of *B. carinata* has a typical quadripartite organization and is 153,641 bp in length. The genome is composed of a large single-copy region (LSC, 83,561 bp), a small single-copy region (SSC, 17,696 bp), and two inverted repeats (IR, 26,192 bp). GC content of the genome is 36.39%. A total of 113 unique genes were predicted, including 79 were protein coding, 4 rRNA, and 30 tRNA genes. Among these genes, 18 genes were duplicated (7 tRNAs, 4 rRNAs, and 7 protein coding genes). Sixty-eight SSR loci, including 11 compound SSRs, were identified in this cp genome by MISA (Beier et al. 2017).

The phylogenetic tree was constructed based on complete cp sequences of *B. carinata* and other 30 related species in the Brassicaceae, and two *Aethionema* species were designated as the outgroup. These sequences were aligned using the default settings in MAFFT version 7 (Katoh and Standley 2013). The tree was constructed with the MEGA7.0 (Kumar et al. 2016) using maximum-likelihood (ML) optimality criterion with the Tamura-Nei nucleotide substitution model. The bootstrap analysis with 1000 replicates was used to confirm the stability of each tree node. *B. carinata* and *B. nigra* were fully resolved on a branch sister to other species of *Brassica* (Figure 1). This result was consistent with the work of Li et al. (2017), supporting the idea that the cytoplasm of *B. carinata* derived from *B. nigra*. The assembly of chloroplast genome sequence of *B. carinata* will be helpful for further study on its phylogenetic study, population genetics and breeding.

**Figure 1.**
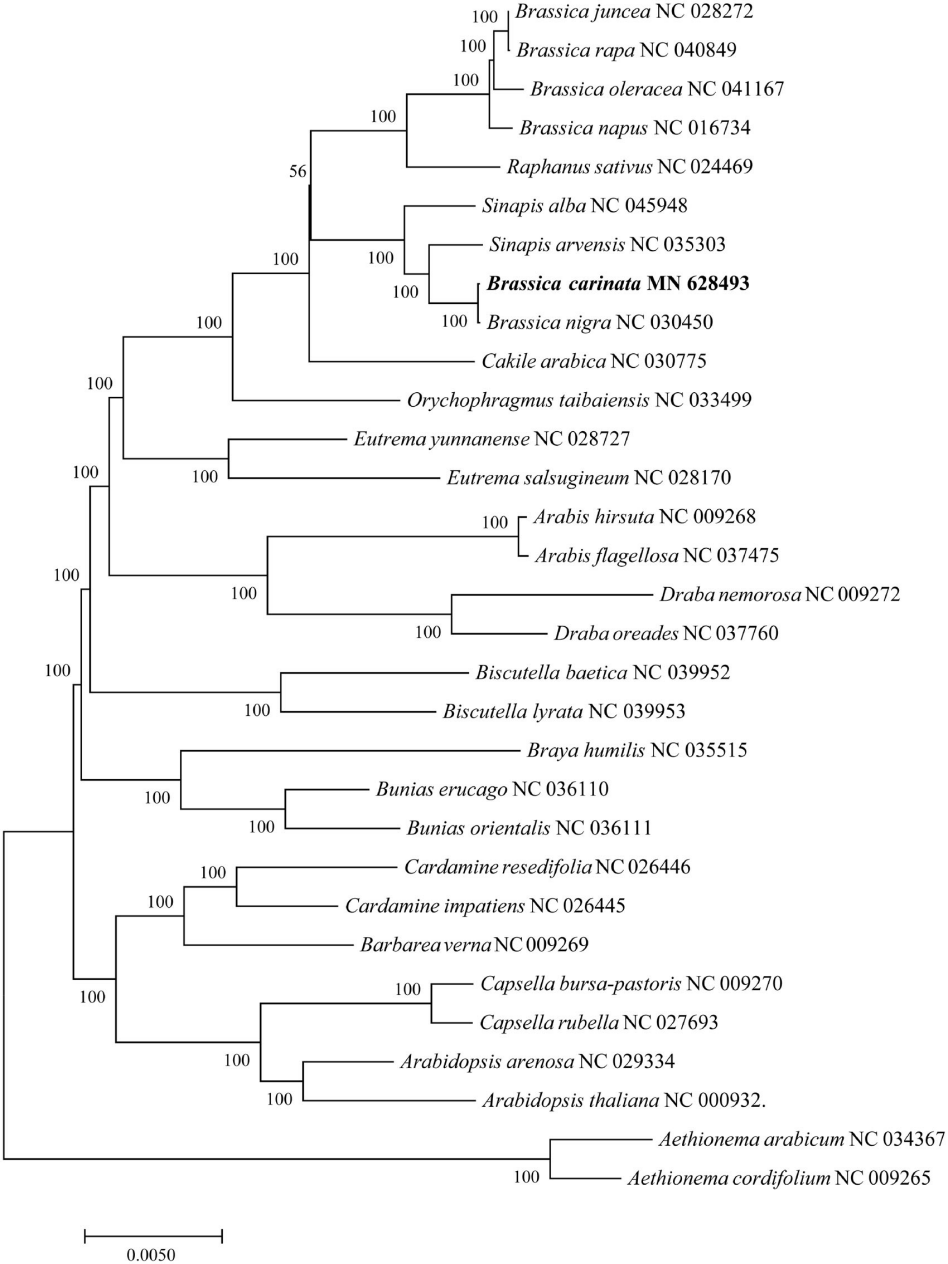
Maximum-likelihood phylogenetic tree inferred from 31 complete chloroplast genome sequences. The position of *B. carinata* is marked in bold and bootstrap values are listed for each branch.

## Data Availability

The data that support the findings of this study are openly available in GenBank of NCBI at [https://www.ncbi.nlm.nih.gov] (https://www.ncbi.nlm.nih.gov/) under the accession no. MW628493. The associated BioProject, SRA, and Bio-Sample numbers are PRJNA721808, SRR14235359, and SAMN18739861 respectively.
